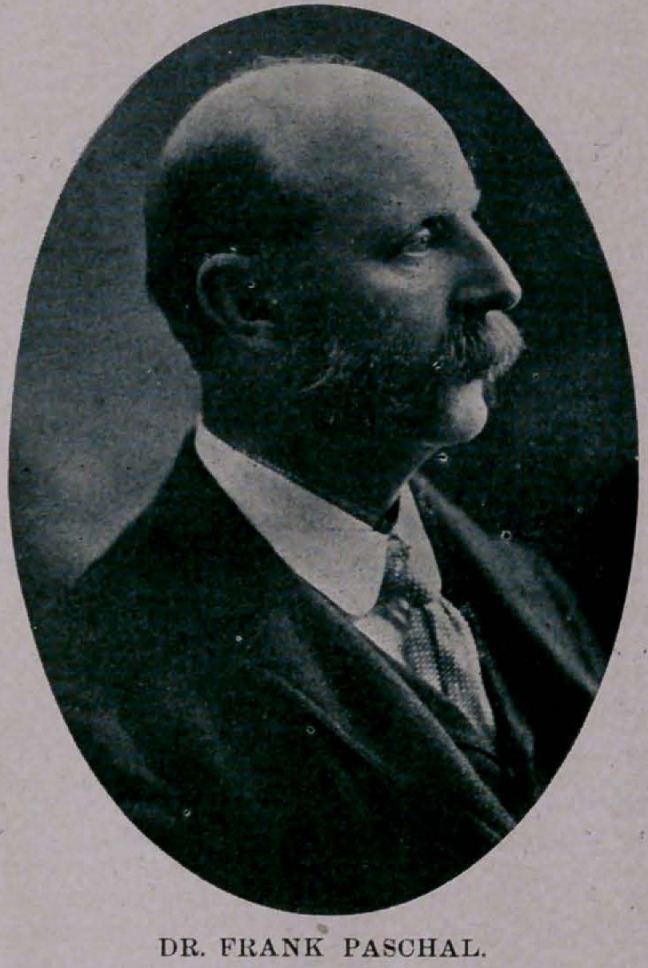# Dr. Frank Paschal, President State Medical Association

**Published:** 1903-05

**Authors:** 


### DR. FRANK PASCHAL, PRESIDENT STATE MEDICAL ASSOCIATION.

Dr. Paschal was born in San Antonio, Texas, October 22, 1849; received a common school education in that city; began the study of medicine under Dr. Geo. Cupples in 1868; graduated from the Louisville Medical College in 1873, and was awarded the prizes for the best thesis for Principles and Practice of Medicine, and for Materia Medica and Therapeutics. He was elected Interne to the Louisville City Hospital in 1873, after a competitive examination; served one year in the hospital. He went to Mexico after his term in the hospital expired, and upon his arrival at the town of Presidio, Mexico, was appealed to to remain there and practice during a raging epidemic of virulent small-pox. He remained and rendered such service to the stricken community as it was possible for him to do, there being no medical aid within 100 miles of the place. After the epidemic subsided, he went to Chihuahua, Mexico, and four days after his arrival, went before the State Board of Medical

Examiners and passed a medical examination in the Spanish language, for licence to practice medicine. The certificate was unanimously granted. The examination was rigid and thorough and Dr. Pachal was the second foreigner who passed the State Board of Examiners in that State. Shortly afterwards he was appointed physician to the City Hospital of Chihuahua, Mexico, and had charge of the same fourteen years. After the completion of the Mexican Central Railroad in Mexico, he was made chief surgeon of

DR. FRANK PASCHAL.

the road, and organized the Medical Department of the Mexican Central Railroad, and held the position of chief surgeon for seven years, and up to the time that he left Mexico to return to his native city in 1892. He was elected, in 1893, President of the West Texas Medical Association. In 1898 was appointed President of the Board of Health of the City of San Antonio. Served as City Health Officer in that city for four years, from 1899 to 1903, and had charge of the City Hospital during that time. He was appointed a member of the State Board of Examiners for the State of Texas in 1891 and served the alloted term of two years. (First Board of Medical Examiners that was appointed by the State of Texas.) He now holds the positions of Second Vice President of the American Congress on Tuberculosis, Third Vice President of the Southwestern Tri-State Medical Society of Texas, Oklahoma and Indian Territory.

Dr. Paschal has contributed several very valuable papers to the *Texas Medical Journal* and the "Transactions," notably one (illustrated) on Lithotomy, in the June, 1900, number. The Doctor has always taken an active interest in medical affairs, and in the upbuilding of the medical profession. We present herewith a cut from latest photo.

## Figures and Tables

**Figure f1:**